# ICDW-YOLO: An Efficient Timber Construction Crack Detection Algorithm

**DOI:** 10.3390/s24134333

**Published:** 2024-07-03

**Authors:** Jieyang Zhou, Jing Ning, Zhiyang Xiang, Pengfei Yin

**Affiliations:** 1 College of Computer Science and Engineering, Jishou University, Jishou 416000, China; 2021404285@stu.jsu.edu.cn (J.Z.); sbxzy@foxmail.com (Z.X.); 2 School of Communication and Electronic Engineering, Jishou University, Jishou 416000, China; 2021404385@stu.jsu.edu.cn

**Keywords:** neural network, object detection, crack detection

## Abstract

A robust wood material crack detection algorithm, sensitive to small targets, is indispensable for production and building protection. However, the precise identification and localization of cracks in wooden materials present challenges owing to significant scale variations among cracks and the irregular quality of existing data. In response, we propose a crack detection algorithm tailored to wooden materials, leveraging advancements in the YOLOv8 model, named ICDW-YOLO (improved crack detection for wooden material-YOLO). The ICDW-YOLO model introduces novel designs for the neck network and layer structure, along with an anchor algorithm, which features a dual-layer attention mechanism and dynamic gradient gain characteristics to optimize and enhance the original model. Initially, a new layer structure was crafted using GSConv and GS bottleneck, improving the model’s recognition accuracy by maximizing the preservation of hidden channel connections. Subsequently, enhancements to the network are achieved through the gather–distribute mechanism, aimed at augmenting the fusion capability of multi-scale features and introducing a higher-resolution input layer to enhance small target recognition. Empirical results obtained from a customized wooden material crack detection dataset demonstrate the efficacy of the proposed ICDW-YOLO algorithm in effectively detecting targets. Without significant augmentation in model complexity, the mAP50–95 metric attains 79.018%, marking a 1.869% improvement over YOLOv8. Further validation of our algorithm’s effectiveness is conducted through experiments on fire and smoke detection datasets, aerial remote sensing image datasets, and the coco128 dataset. The results showcase that ICDW-YOLO achieves a mAP50 of 69.226% and a mAP50–95 of 44.210%, indicating robust generalization and competitiveness vis-à-vis state-of-the-art detectors.

## 1. Introduction

Since ancient times, wooden materials have been extensively utilized due to their practicality, renewability, and eco-friendly properties, resulting in the construction of numerous historically and aesthetically significant ancient buildings. Statistics reveal that over 70% of ancient structures in China feature wooden frameworks [1]. Over time and with environmental changes, ancient buildings have undergone various forms of deterioration, with cracks emerging as the most prevalent issue. The causes of such cracks within wooden structures are multifaceted and deleterious, often exacerbated by financial constraints and limitations in repair techniques, thereby significantly impeding the sustainable development of wooden architectural components within ancient structures, ultimately leading to structural collapse in certain instances. Consequently, preservation efforts and research endeavors focusing on protective measures for wooden architectural heritage have garnered considerable attention.

Within wooden structure inspections, non-destructive testing methodologies play a pivotal role in defect identification within architectural constructions. Non-destructive testing methodologies offer enhanced efficiency and reduced destructiveness compared to conventional measurement techniques, encompassing methodologies such as laser scanning [2], ultrasonic testing [3], and visual inspections. Among these methodologies, instruments reliant on ultrasonic and electromagnetic waves exhibit limited detection ranges and fewer discernible categories. At the same time, vision-based non-contact methodologies boast superior detection speeds, real-time performance, and accuracy. Recently, vision-based non-destructive testing technologies have witnessed a burgeoning promotion, successfully facilitating crack detection on metallic surfaces in industrial settings, thereby markedly decreasing operational costs vis-à-vis alternative detection methodologies. Furthermore, the advent of deep learning (DL) has elicited favorable responses in crack detection research, with DL object detection technologies gradually permeating the domain of non-destructive testing within wooden structures [4,5,6,7,8]. Present research predominantly harnesses convolutional neural networks and YOLO series models to discern and categorize wooden architectural sites [9,10], wood, and wooden utensils [11,12] by extracting texture features and analysis of color attributes.

The identification and localization of cracks within wooden edifices pose significant challenges attributable to the multifarious causes and diverse array of damage types, notably the disordered distribution of cracks. The principal obstacles encountered in crack detection via DL include reconciling model complexity with accuracy. Key issues in the design of detection models encompass excessively intricate architectures, suboptimal detection rates for minute cracks, and stringent criteria about the quality of input data sources [13,14,15].

This study explores an optimized detection model, to augment the efficacy of DL-based object detection technology in the non-destructive assessment of wooden structures for crack detection. The YOLO series detectors are characterized as single-stage object detectors [16], notable for their exceptional processing speed and real-time performance compared to the two-stage detectors within the CNN series.

Consequently, this paper proposes a lightweight object detection algorithm based on the improved YOLOv8 [17], designed for crack detection in wooden materials. We initiate by designing a feature optimization module composed of GSConv and GS bottleneck [18] to enhance feature fusion and processing capabilities. Within this framework, GSConv emerges as a pivotal component capable of significantly reducing the model’s complexity while preserving its efficacy. Within the refined YOLO network architecture, modifications are made to the neck network, drawing upon the principles of the gather–distribute (GD) mechanism [19], aimed at bolstering the capacity for feature fusion. Concurrently, the retention of small target features is ensured by establishing connections between layers with heightened sampling rates, thereby amplifying the precision in identifying minute crack targets within the YOLO network. Moreover, the convergence speed was further enhanced by incorporating Wise-IoU [20], featuring a dual-layer attention mechanism and dynamic gradient gain characteristics. Lastly, a dataset specifically tailored for crack detection in wooden materials was curated.

The primary contributions of this study can be delineated as follows:

- We propose a feature optimization module composed of GSConv and GS Bottleneck that enhances feature fusion and processing capabilities and balances the price and performance of the algorithm.
- We designed the network architecture using the GD mechanism, incorporating autonomously designed modules. This redesign notably enhances the information fusion capability of the neck network without significantly increasing model complexity, thereby efficiently preserving the features of small targets.
- We introduce a Wise-IoU with a dual-layer attention mechanism and dynamic gradient gain characteristics. This introduction accelerates the convergence speed of the model and enables it to adapt to the varying data quality in the dataset.

## 2. Related Works

Image-based crack detection in wooden materials stands as a multifaceted research domain, integrating disciplines such as image processing, pattern recognition, and DL. Presently, this field exhibits a categorical division into two principal classifications which are crack detection in wooden materials founded on digital image processing methods and crack detection based on DL methodologies. Crack detection approaches originating from digital image processing methods manifest an earlier inception and boast a comparatively mature technological framework. Conversely, methods reliant on DL surfaced at a later juncture, demonstrating heightened potential for advancement.

### 2.1. Traditional Methods for Wood Crack Detection

Wooden material crack detection constitutes a research domain focused on discerning cracks within wooden materials, grounded in traditional digital image processing. This study employs algorithms of digital image processing to perform feature extraction, thereby contributing to determining the presence of cracks in images or videos. Conventional methodologies for target detection involve introducing the integral image for swift feature computation, proposing the AdaBoost algorithm, and implementing the cascade structure by Viola-Jones [21], utilizing the histogram of oriented gradients (HOG) for facile capture of local shape features [22,23], and employing the deformable part-based model as an extension of HOG, especially adept at detecting occluded objects [24,25]. Meanwhile, some swarm intelligence algorithms, such as harmony search [26,27], cuckoo search [28,29], and sparrow search [30] are widely used to optimize the performance of the detection results.

The scale-invariant feature transform algorithm [31] is utilized extensively in the realm of computer vision for object identification and definition by leveraging local image features. Its applicability extends notably to the task of matching objects or scenes across disparate viewpoints. As the imperatives for heightened precision and computational efficiency escalate within this domain, there exists a persistent drive toward refining existing methodologies. This endeavor manifests in the continuous enhancement of various detection techniques, exemplified by advancements such as the Oriented FAST and Rotated BRIEF (ORB) [32], which offer superior efficiency in contrast to the conventional SIFT approach. Furthermore, contemporary research endeavors have delved into the integration of hybrid models as a means of augmenting the efficacy of target detection processes [33].

### 2.2. CNN-Based Methods for Wood Crack Detection

Traditional wooden material crack detection, based on digital image processing, encounters significant challenges in extracting complex semantic information related to cracks. These challenges often result in increased rates of false positives and false negatives. The rapid advancement of DL has facilitated the widespread adoption of neural network-based DL methods across various domains. These methods are notably applied in solving equations [34,35,36], performing matrix computations [37,38], and enhancing intelligent control systems [39,40]. In numerous DL models, convolutional neural networks (CNNs) exhibit excellent self-learning capabilities and can adeptly reveal profound semantic insights within images. With the continuous evolution of CNNs and the emergence of high-performance computing devices, areas such as image classification and object detection have witnessed substantial development. This progress has led to the successive introduction of efficient single-stage object detection networks, including YOLO [16], single-shot multi-box detectors [41], and efficient object detection.

Moreover, in the realm of DL, backbone networks serve as feature extractors for detectors, encompassing AlexNet [42], ResNet [43], DenseNet [44], MobileNet [45], ShuffleNet [46], and ShuffleNetV2 [47], among others. There is ongoing research into lightweight network structures [48] to achieve computational acceleration and conserve computational resources.

As a consequence of these advancements, object detection methods rooted in DL have found widespread adoption in the field of wooden material crack detection. The fundamental process can be divided into two stages: training and inference. During the training stage, the initial steps involve collecting a large number of images relevant to wooden material cracks to construct a comprehensive training dataset. Subsequently, data are annotated or preprocessed to adapt to the specific task. Following this, the dataset undergoes preprocessing to enhance data diversity. The processed data are then used as input for the CNN for feature extraction. Finally, features are classified, and this sequential step is iteratively performed during the training process to obtain an optimal model.

## 3. Methodology

### 3.1. YOLOv8 over Review

The YOLO model represents an object recognition and localization algorithm grounded in deep neural networks. Its most prominent attribute lies in its high-speed operational capabilities, rendering it particularly suited for real-time systems. This model has garnered significant acclaim and has been extensively employed in the domain of computer vision. YOLOv8 [17] distinguishes itself from its forerunners, such as YOLOv5 [49] and YOLOv7 [50]; it now claims the position of the most innovative computer vision model globally and stands as a highly adaptable platform for customization. The network’s fundamental structure primarily encompasses three key components: the head, backbone, and neck.

In YOLOv8, the enhanced CSPDarknet53 [51] functions as the backbone network, facilitating the generation of five discrete scale features through a sequence of five successive downsampling stages. Furthermore, a seminal design innovation within the YOLO framework is the incorporation of the spatial pyramid pooling (SPP) structure [52]. Inspired by the path aggregation network (PANet) [53], the PAN-FPN architecture is integrated into the neck component of YOLOv8. A comprehensive network structure that harmonizes both top-down and bottom-up components is created in this approach, which through feature fusion blends surface-level positional insights with deep semantic details, thereby enriching the breadth and depth of features.

Within the head structure of the YOLOv8, it embraces a ‘decoupled head’ and incorporates the distribution focal loss (DFL) [54] concept for object classification and bounding box regression predictions. This version refines loss functions, using the vertical federated learning (VFL) [55] loss for classification and the complete intersection over union (CIoU) [56] loss alongside the DFL for regression, each offering distinct features. In addition, anchor-free detection of the YOLOv8 simplifies distinguishing between positive and negative samples, and the task-aligned one-stage object detection (TOOD) [57] is integrated for better sample allocation, which improves both the robustness and detection accuracy of the model.

### 3.2. Improve the Structure of ICDW-YOLO

#### 3.2.1. The Framework of ICDW-YOLO

Notwithstanding the incorporation of feature pyramid networks (FPNs) [58] and PANet [53] in the current YOLO algorithm for multi-scale feature fusion, a fusion issue persists concerning feature information. Specifically, the fundamental YOLO series algorithms adopt a conventional FPN structure within the neck network segment. Traditional FPN structures are constrained to fully fuse features solely from adjacent layers, with information from other layers being attainable only through indirect recursive methods. This results in escalated algorithmic complexity and a consequential loss of transmitted information. The employed transmission mode may, during computation, lead to a substantial loss of information as the interaction between non-adjacent layers relies solely on the selection of information through intermediate layers, consequently leading to the loss of specific information. Consequently, the information from one layer may effectively aid only adjacent layers, thereby diminishing its support for other non-adjacent layers. Thus, the overall efficacy of information fusion may be restricted.

In light of this, there arises a necessity to introduce the GD mechanism, which systematically gathers and amalgamates information from diverse scales through a unified module, subsequently distributing the fused features across distinct layers [19]. Within this mechanism, the feature alignment module (FAM) [59] and information fusion module (IFM) synergistically aggregate features from disparate hierarchical echelons. The inject module subsequently disburses the amalgamated information throughout diverse layers of the network. This strategic orchestration enables the model to judiciously harness multi-scale features, thus elevating object detection precision while retaining low latency. This approach not only circumvents the inherent information loss observed in traditional FPN structures but also amplifies the information fusion capacity of the neck section without a significant increase in latency. It more judiciously harnesses the features extracted by the backbone network.

**Figure 1:**
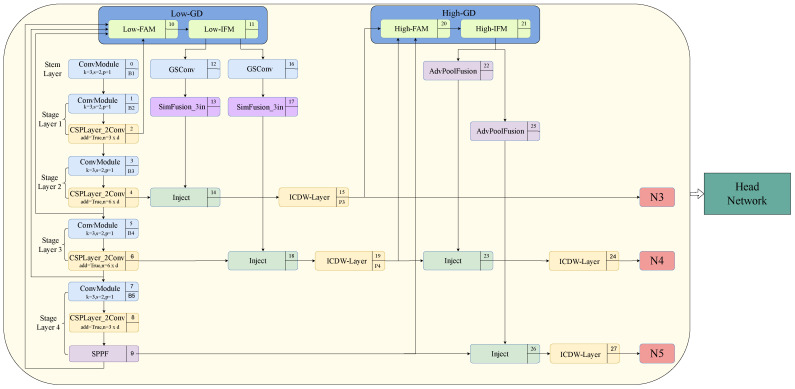
Network structure of ICDW-YOLO.

Specifically, as elucidated in Figure 1, this study introduces the GD mechanism into the neck part of the original YOLOv8 network. The low-GD is utilized to replace the upsampling fusion stage of PANet, while the high-GD replaces the downsampling fusion stage of PANet. Moreover, the introduction of a higher-resolution B2 layer into low-GD aims to preserve the feature information of small targets as comprehensively as possible. Within low-GD, using the B4 layer as the reference, large feature maps such as the B2 layer and B3 layer undergo downsampling through average pooling, whereas small feature maps like the B5 layer undergo upsampling using bilinear interpolation to standardize the feature map sizes, after which the merged features are acquired. Subsequently, the features obtained through low-GD fusion, namely P3 layer, P4 layer, and P5 layer, undergo feature fusion via high-GD. This procedure effectively enhances information fusion performance and preserves the information features of small targets.

Moreover, within the domain of object detectors, the neck serves as a crucial intermediary between CNN’s backbone network and the head network. This facilitates the fusion and processing of features, thereby enhancing the precision and efficiency of detection. In order to effectively balance the model’s performance with its complexity, this study has developed a new module based on the GS Bottleneck framework. This module replaces the CSP Bottleneck with a two-convolution (c2f) model in the neck network and is named the ICDW layer model. Together with the introduction of GSConv, this addition further enhances the cost-effectiveness of model computation.

Therefore, the combination of the GD mechanism, ICDW layer, and GSConv collectively constitute the network architecture optimization of ICDW-YOLO.

#### 3.2.2. ICDW Layer

**Figure 2:**
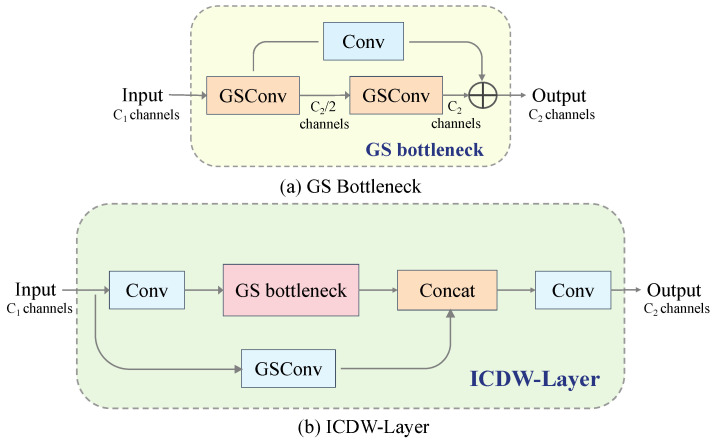
Structure of the GS bottleneck module and ICDW layer.

The ICDW layer is primarily designed to replace the c2f module within the original neck network, thereby effectively facilitating feature fusion and processing while also balancing the model’s performance and computational cost. As depicted in Figure 2, the ICDW layer primarily incorporates the GSConv and GS Bottleneck module, which is constructed by stacking GSConv, to enhance the network’s feature extraction capabilities.

The integration of GSConv addresses the prevalent computational speed challenge in CNN predictions. With each instance of spatial compression and channel expansion in the feature map, there is a consequential partial loss of semantic information. Sparse convolution (SC) [60] maximally retains implicit connections among channels, whereas depthwise separable convolution (DSC) [61] completely severs these connections. GSConv strives to preserve these connections to the utmost extent possible. The design and implementation of this module effectively enhance the network’s feature fusion capability while reducing computational complexity and inference time.

#### 3.2.3. Wise-IoU

In order to effectively address the negative impact of low-quality data in the training set on the model’s generalization ability, we introduce Wise-IoU [20] in ICDW-YOLO, incorporating a dual-layer attention mechanism constructed based on metrics.

IoU serves as a metric for quantifying the degree of overlap between the predicted box and the ground truth in the target detection task. In this context, $W_{i}$ and $H_{i}$ represent the height and width of the intersection area respectively, while $S_{u}$ denotes the area union. The gradient computation of $L_{IoU}$ in backpropagation is elucidated in Equation (2). In such cases, in the absence of overlap between the bounding boxes, i.e., $w_{i}=0$ or $H_{i}=0$, the gradient of $L_{IoU}$ in backpropagation becomes non-existent, thus the width cannot be updated during training.

(1) $$L_{IoU}=1-IoU=1-\frac{W_{i}H_{i}}{S_{u}}$$

(2) $$\frac{\partial L_{IoU}}{\partial W_{i}}=\left\{\begin{array}{c} -H_{i}\frac{IoU+1}{S_{u}},W_{i}>0\\ 0,W_{i}=0 \end{array}\right.$$

In addressing this issue, Wise-IoU separates $W_{g}$ and $H_{g}$ according to the paradigm in Equation (3), thus constructing the penalty term $R_{WIoU}$ as shown in Equation (4). This term significantly amplifies the $L_{IoU}$ for ordinary quality anchor boxes and effectively prevents $R_{WIoU}$ from impeding gradient convergence. This process weakens the penalty of geometric metrics when there is a good overlap between anchor and target boxes, without excessive interference in training, thereby improving the model’s generalization ability.

(3) $$L_{i}=L_{IoU}+R_{i}$$

(4) $$R_{WIoU}=\exp\left(\frac{{\left(x-x_{qt}\right)}^{2}+{\left(y-y_{gt}\right)}^{2}}{{\left(W_{g}^{2}+H_{g}^{2}\right)}^{*}}\right),R_{WIoU}\in \left[1,e\right)$$

Moreover, to enhance the regression of bounding boxes and mitigate harmful gradients arising from low-quality training data, we introduce an outlier assessment β to evaluate the quality of anchors, as demonstrated in Equation (5), and assign smaller gradient gains to anchors with larger outliers. Ultimately, this results in the realization of a Wise-IoU, which not only introduces a non-monotonic focusing coefficient but also incorporates a dual-layer attention mechanism attentive to distance, as demonstrated in Equations (6) and (7).

(5) $$\beta =\frac{L_{L_{oU}}^{*}}{\bar{L_{IoU}}},\beta \in \left[0,+\infty \right)$$

(6) $$r=\frac{\beta }{\delta \alpha^{\beta -\delta }}$$

(7) $$L_{WIoU}=rR_{WIoU}L_{IoU}$$

In the scenario where β=δ, δ results in r=1, and if the outliers of the anchor boxes satisfy β=C, *C* is the quality partition standard of the anchor boxes, the anchor boxes gain the highest gradient. Moreover, since $\bar{L_{IoU}}$ is dynamic and the quality partition standard of the anchor boxes is also dynamic, a gradient gain allocation strategy that best fits the current situation occurs at every moment, thus further accelerating the convergence of the model and improving its performance.

## 4. Experimental Results

### 4.1. Dataset Preparation and Augmentation

The dataset in this study primarily consists of three components. Firstly, an original dataset for the detection of cracks in wooden materials, to evaluate the model’s performance in this specific domain. Secondly, a dataset intended to assess the model’s generalization capability. This dataset includes a dynamic fireworks detection dataset as well as an aerial image object detection dataset, covering various small targets and targets with dynamic features. Finally, a publicly available dataset is employed for standardizing the evaluation of the model, with the coco128 dataset being selected.

#### 4.1.1. Crack Dataset

Typically, datasets are procured through online searches; nevertheless, the scarcity and heterogeneity of these images are noteworthy. Specific image categories boast publicly accessible datasets characterized by superior quality in comparison to their counterparts. Furthermore, the predominant approach entails the acquisition of images through on-site photography. Owing to the dearth and substandard quality of online images portraying cracks in wooden materials, on-site photography was selected as the preferred methodology. Images capturing fissures in wooden materials for this study were culled from enduring wooden structures dispersed across diverse locales in Hunan Province, China, with a standing history exceeding thirty years. These images were systematically captured from diverse perspectives within environments mirroring natural conditions, encompassing both interior and exterior settings. Diligent efforts were exerted to circumvent excessively shadowed angles and environments.

Due to the necessity of sourcing images of cracks in wooden materials from ancient buildings predominantly constructed of wood, the collection process is inherently challenging, resulting in a limited sample size. Consequently, the initial dataset comprises only 506 images depicting cracks in wooden materials. Given the prevailing uniformity in architectural styles and wood varieties among wooden structures within the same locale, the preliminary screening rigorously eliminated excessively blurred and redundant images. This process culminated in a refined dataset of 406 images, thus affirmatively ensuring dataset quality.

**Figure 3:**
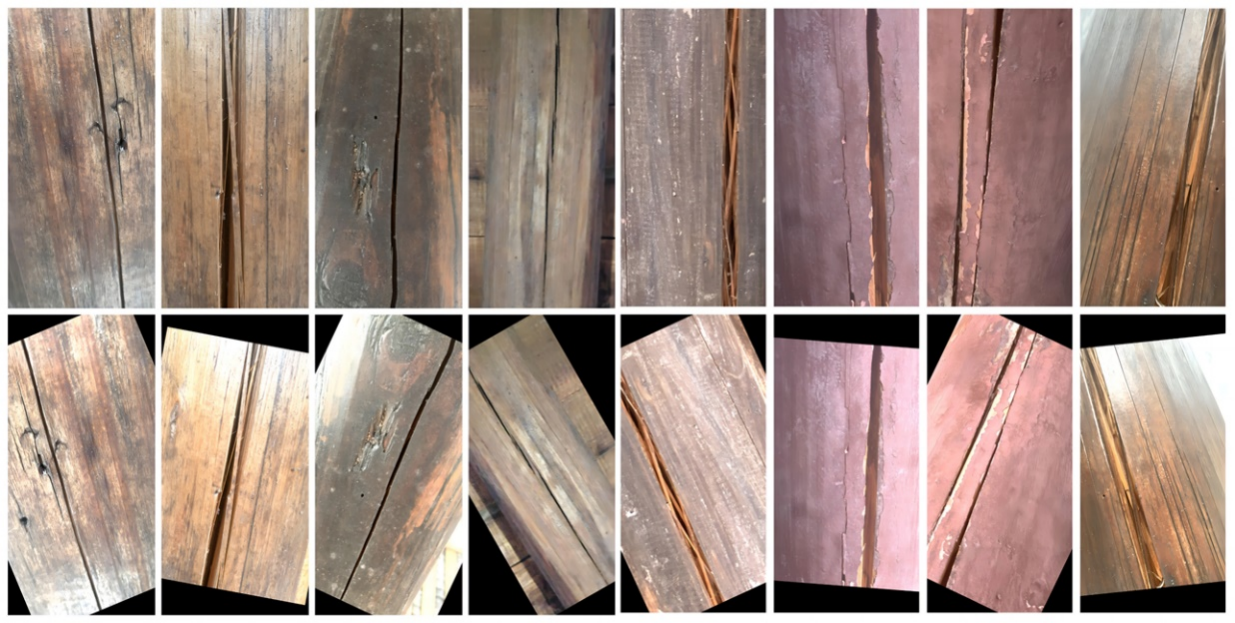
Example of feature enhancement.

After the initial collection, the images underwent meticulous annotation using the labeling tool. Moreover, owing to the constrained dimensions of the initial dataset, imperative measures encompassing feature augmentation methodologies were instituted to refine image processing, thereby substantively amplifying and elevating its quality. The data augmentation methodology employed in this study adopts a comprehensive framework integrating rotation, blurring, luminosity adjustment, image stretching, and the infusion of Gaussian noise. Post application of feature augmentation, the dataset encompassed a cumulative total of 1617 different images; the effect is shown in Figure 3. As delineated in Table 1, the dataset was partitioned into training, validation, and test subsets, adhering to a proportion of 7:2:1.

#### 4.1.2. Other Dataset

This study introduces additional datasets to assess the model’s performance, generalization capabilities, and applicability in various detection scenarios. A significant concern addressed is the limited diversity and scale within the wooden material dataset, which restricts comprehensive testing of the model’s multifaceted capabilities. While the wooden material crack detection dataset includes both single-object and multi-object detection, it suffers from limited scale and targets that lack dynamic characteristics and substantial scale variation. In contrast, the fire and smoke detection dataset comprises dynamic targets, enabling a thorough evaluation of the ICDW-YOLO model’s proficiency in detecting dynamic objects. This assessment primarily focuses on scenarios where targets display dynamic features, influenced by the mobile nature of embedded platforms during development.

The initial dataset introduced is the Fire and Smoke dataset, as delineated in Table 2, comprising two distinct components. One segment emanates from the publicly available wildfire smoke dataset provided by the State Key Laboratory of Fire Science (SKLFS), University of Science and Technology of China (USTC) [62]. The second segment constitutes a dataset meticulously collected and annotated during our research, denoted as the Fire and Smoke dataset in subsequent discussions.

Furthermore, the DOTAv2-tiny dataset is utilized to evaluate the ICDW-YOLO model’s capacity to identify small and multi-scale targets, especially in contexts demanding multi-object recognition. Given that the Crack dataset is characterized as a single-target dataset with relatively sizable targets, the introduction of the DOTA dataset becomes imperative, encompassing both multi-object and small-object recognition. The DOTA dataset, designed for object detection in aerial images [63], is an extensive collection acquired from diverse platforms and sensors. The DOTAv2 version, presently available in three versions, was selected for experimentation, and given the considerable magnitude of the DOTAv2 dataset, a judiciously chosen subset is employed in this research without compromising the dataset’s integrity. The chosen dataset size is detailed in Table 2, denoted as DOTAv2-tiny in subsequent discussions. Finally, a publicly available dataset is used for standardizing the evaluation of the model, with the coco128 dataset selected, as shown in Table 2.

### 4.2. Training Parameters and Experimental Environment

In order to ensure methodological robustness, all experiments were rigorously executed under uniform hardware conditions. The experimentation was conducted employing a custom-assembled computer system, which features specific and finely detailed specifications, meticulously elucidated in Table 3, inclusive of an Nvidia GeForce 4060 Ti graphics processing unit endowed with 16 GB of memory (Nvidia, Santa Clara, CA, USA), supplemented by 32 GB of RAM, and powered by an Intel Core i7 processor 14,700 K running at 5.6 GHz across 20 cores (Intel, Santa Clara, CA, USA).

Setting hyperparameters is pivotal for shaping model performance and ensuring the success of algorithmic enhancements. In the process of refining the YOLOv8 model, it is imperative to uphold uniform hyperparameter configurations. This consistency serves as a linchpin, affirming the efficacy of model advancements and expediting precise performance appraisals conducted both before and after enhancements. The act of adjusting hyperparameters during algorithmic refinement introduces the potential to obscure the origin of performance variations—whether they emanate from the inherent enhancements of the algorithm or result from alterations in the hyperparameters. Consequently, in order to ascertain an unambiguous and rigorous assessment of advancements, this paper steadfastly adheres to a standardized set of hyperparameters, as shown in Table 4.

### 4.3. Evaluation Metrics

In the empirical evaluation of performance metrics, the study incorporated the utilization of average precision (AP), F1 score, and accuracy. The computation of accuracy entails the establishment of a ratio, specifically the division of true positives (TPs) and true negatives (TNs) by the aggregate of identified samples, as explicated in Equation (8). What is noteworthy here is that TPs denote instances correctly predicted as positive, while TNs designate instances accurately predicted as negative. Importantly, false positives (FPs) constitute instances erroneously predicted as positive, and false negatives (FNs) represent instances genuinely positive but erroneously predicted as negative.

(8) $$Accuracy=\frac{\left|TP\right|+\left|TN\right|}{\left|TP\right|+\left|TN\right|+\left|FP\right|+\left|FN\right|}$$

Precision and recall have emerged as pivotal metrics in the appraisal of a classification model’s efficacy, particularly in scenarios wherein achieving equilibrium between true positives and false positive predictions is imperative. Precision, delineated in Equation (9), quantifies the accuracy of the model’s positive prognostications. It manifests as the quotient of TPs divided by the summation of TPs and false positives (FPs). Conversely, recall, as articulated in Equation (10), gauges the model’s acumen in identifying true positives from the entire pool of actual positive instances. This is computed as the ratio of TPs to the sum of TPs and false negatives (FNs). Heightened recall underscores the model’s proficiency in capturing a substantial proportion of actual positives. Both metrics are of paramount significance in comprehending the model’s efficacy, with precision affording a nuanced focus on the accuracy of positive predictions, and recall accentuating the entirety of positive instances captured.

(9) $$Precision=\frac{\left|TP\right|}{\left|TP\right|+\left|FP\right|}$$

(10) $$Recall=\frac{\left|TP\right|}{\left|TP\right|+\left|FN\right|}$$

Average precision (AP), elucidated in Equation (11), serves as a metric for appraising the model’s capability to accurately categorize objects and sequence them predicated on predicted confidence, conventionally summarized as the mean average precision (mAP) across multiple categories, elucidated in Equation (12). F1 score, as delineated in Equation (13), assumes the role of a harmonic mean, encapsulating precision and recall, thereby furnishing a balanced singular metric catering to false positives and false negatives, particularly salient in scenarios where each error type exerts a substantial impact or in datasets characterized by imbalance. Intersection over union (IoU), explicated in Equation (14), quantifies the precision of object localization by assessing the ratio of the overlapping area between predicted bounding boxes (BDs) and ground truth bounding boxes (BGTs) relative to their union area. Cumulatively, these metrics proffer a holistic evaluation of the object detection model’s capacities, spanning both classification accuracy and object localization precision.

(11) $$AP=\int_{0}^{1}p\left(r\right)dr$$

(12) $$mAP=\frac{1}{m}\sum_{i=1}^{m}AP_{i}$$

(13) $$F1Score=2\times \frac{Precision\times Recall}{Precision+Recall}$$

(14) $$IoU=\frac{\left|B_{D}\cap B_{GT}\right|}{\left|B_{D}\cup B_{GT}\right|}$$

FLOPs (floating point operations) serve as indicators for assessing algorithmic complexity and are commonly used indirectly to gauge the computational speed of neural network models. In the context of convolutional layers, the formula for calculating FLOPs is depicted in Equation (15), where $C_{\mathrm{in}}$ denotes the number of channels in the input tensor of the convolutional layer, $C_{\mathrm{out}}$ represents the number of channels in the output tensor, and *K* indicates the size of the convolutional kernel. Higher FLOP values typically indicate that the model exhibits a more intricate architecture or necessitates greater computational resources for processing input data, thereby providing a metric to quantify the complexity of the model.

(15) $$\mathrm{FLOPs}=2HW\left(C_{\mathrm{in}}K^{2}+1\right)C_{\mathrm{out}}$$

In the evaluation of the model’s inference and recognition speeds, this study adopts FPS (frames per second) as the principal metric, as depicted in Equation (16), where $t_{p}re$ represents the pre-processing time, $t_{i}nf$ represents the inference time, and $t_{p}ost$ represents the post-processing time. FPS denotes the rate at which the network processes frames per second, indicating either the number of images processed per second or the time required to process a single image, thereby assessing detection speed. A shorter processing time correlates with higher speed.

(16) $$FPS=\frac{1s}{t_{p}re+t_{i}nf+t_{p}ost}$$

### 4.4. Experimental Results and Analysis

#### 4.4.1. Quantitative Comparison and Evaluation

The quantitative evaluation in this article aimed to comprehensively assess the effectiveness of the proposed methodology. Various metrics, including precision, recall, mAP50, mAP50–95, and so on, were calculated using Equations (8)–(16). To address the diversity of instances in the dataset, spanning different distances and encompassing both small and large areas, systematic testing was conducted across various DL models.

This study primarily focuses on utilizing DL models for the detection of cracks in wooden materials, with a specific emphasis on protecting ancient buildings through crack detection. Following a thorough evaluation of the dataset, YOLOv8 was selected as the primary framework due to its performance and efficiency in swiftly detecting instances of cracks in wooden materials of different sizes and orientations. The proposed crack detection model, built upon YOLOv8, exhibited significant enhancements across multiple performance metrics.

**Figure 4:**
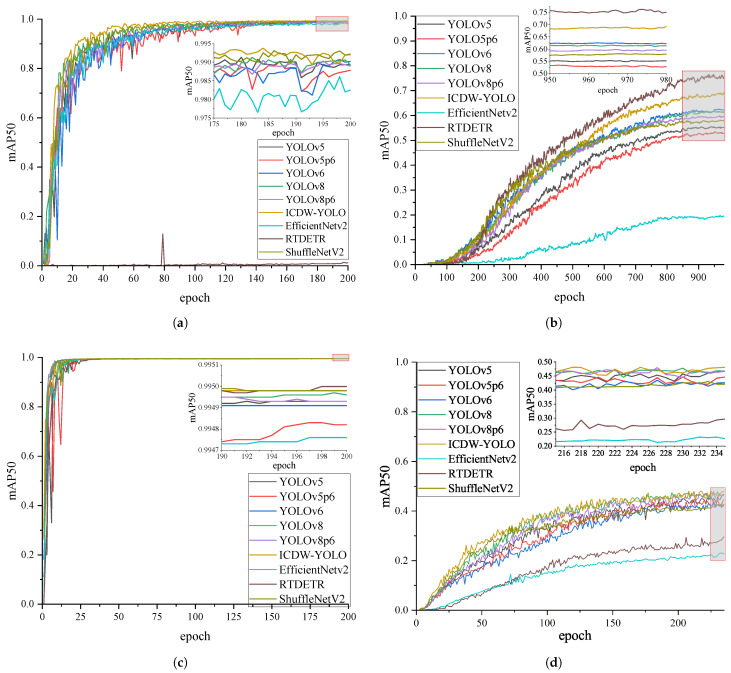
Graph of the change curve of the model evaluation index. (**a**) The mAP50 metric change graph of ICDW-YOLO under the Crack dataset. (**b**) The mAP50 metric change graph of ICDW-YOLO under the coco128 dataset. (**c**) The mAP50 metric change graph of ICDW-YOLO under the Fire and Smoke dataset. (**d**) The mAP50 metric change graph of ICDW-YOLO under the DOTAv2-tiny dataset.

In order to thoroughly evaluate the effectiveness of the proposed method, and in consideration of the challenges in aligning evaluation metrics between traditional detection methods and DL-based detection approaches, this analysis encompasses a range of DL-based object detection technologies and their enhancements, including YOLOv5, YOLOv5p6 [49], YOLOv6 [64], YOLOv8, YOLOv8p6 [17], EfficientNetv2 [65], ShuffleNetV2 [47], and RTDETR [66]. Moreover, in order to assess the model’s generalizability across diverse scenarios and varied requirements, experiments were conducted not solely utilizing the Crack dataset, as mentioned previously, but also incorporating the previously described DATAv2-tiny dataset, Fire and Smoke dataset, and coco128 dataset. The comparative performance analysis between the ICDW-YOLO and various models concerning each dataset is detailed in Table 5, and Figure 4.

The comparative experimental results of the proposed ICDW-YOLO model against various DL-based object detection models on different datasets are presented in Table 5 and Figure 4. In several comparative experiments, RTDETR’s stringent dataset requirements led to poor performance and underfitting on the Crack and DATAv2-tiny datasets. Under identical dataset and training conditions, ICDW-YOLO consistently achieved superior performance metrics, such as precision, recall, and mAP. Specifically, among models of similar complexity, ICDW-YOLO ranked highest for all metrics on the Crack dataset and excelled on datasets like DATAv2-tiny, designed to test detection capabilities in diverse complex environments. It ranked first in precision, recall, mAP50, mAP50–95, and F1 score across multiple datasets.

As detailed in Table 5, on the Crack dataset, ICDW-YOLO maintained inference speed and model complexity without significant increases in FPS or FLOPs, while ranking first in precision, recall, mAP50–95, and F1 score. Its mAP50 was second only to the more complex ShuffleNetV2. On the wooden material detection dataset collected for this study, ICDW-YOLO demonstrated outstanding performance. The model attained precision, recall, mAP50, and mAP50–95 values of 98.756%, 98.013%, 99.201%, and 79.018%, respectively, representing notable enhancements over the original YOLOv8 model.

For more detailed assessments on the DATAv2-tiny and Fire and Smoke datasets, ICDW-YOLO significantly improved performance while maintaining inference speed and without noticeably increasing model complexity. On the DATAv2-tiny dataset, which exhibited significant differences in experimental results, ICDW-YOLO ranked first in precision, recall, mAP50, and F1 score. Finally, on the coco128 dataset, used for a comprehensive evaluation in a small-sample, multi-target environment with scale variations, ICDW-YOLO performed admirably across all five performance metrics. It secured the top position in precision, mAP50, and F1 score, and was second in recall, mAP50, and mAP50–95 only to RTDETR, which has much higher complexity (FLOPs) and lower inference capability (FPS). Compared to the original YOLOv8 model, ICDW-YOLO’s precision increased from 64.601% to 82.128%, recall from 53.456% to 55.181%, mAP50 from 60.256% to 69.226%, and mAP50–95 from 39.627% to 44.210%. Compared to similar models, ICDW-YOLO achieves superior performance with reduced complexity compared to YOLOv6 and attains higher performance without a significant increase in model size compared to the YOLOv8 and YOLOv5 series.

#### 4.4.2. Ablation Study

The ablation experiments are conducted to delineate the contributions and functions of various components within the model, thereby assessing its robustness and performance and providing guidance for optimization and enhancement. This study undertakes a series of ablation experiments aimed at evaluating the effects of the Wise-IoU, and the neck network design method with the GD mechanism, GSConv, and ICDW layer model on the accuracy of detection. Specifically, this research comprises 14 specific ablation experiments on the coco128 and Crack datasets, incorporating the YOLOv8 model, models (a) to (e), and ICDW-YOLO. The detailed findings of these experiments are summarized in Table 6, which extensively assesses the potential enhancements of the YOLOv8 baseline model resulting from these modifications. These assessments are based on various metrics, including precision, recall, mAP50, mAP50–95, and FLOPs.

The ablation study suggests that while the YOLOv8 object detection model demonstrates robust performance, its effectiveness may not achieve its utmost potential under certain circumstances. These findings indicate the potential for enhancing model accuracy across diverse contexts by integrating Wise-IoU, ICDW layer, and GSConv into the YOLOv8 network architecture and adopting the neck network design method with the GD mechanism. Specifically, the introduction of Wise-IoU can effectively enhance various metrics of the model without increasing its complexity, while the introduction of GSConv can reduce model complexity to some extent with minimal impact on performance. However, introducing the ICDW layer or GD mechanism alone would significantly increase the model’s size, leading to a considerable rise in complexity as the cost of performance enhancement.

As demonstrated by the experimental data in Table 6, the introduction of either the ICDW layer or the GD mechanism results in a significant increase in model size while simultaneously enhancing performance. For example, the FLOPs on the coco128 dataset increased from 8.7 GFLOPs in the original YOLOv8 model to 12.5 GFLOPs and 20.0 GFLOPs for models (a) and (b), respectively. Notably, model (b) also incorporates GSConv, which aids in reducing model complexity. Conversely, the GD mechanism introduced in model (a) improves performance without substantially increasing complexity by enhancing multi-scale feature fusion capability. Consequently, integrating the GD mechanism and GSConv can effectively counterbalance the increase in model complexity caused by the ICDW layer while preserving the performance enhancements brought by the GD mechanism and ICDW layer. Additionally, the introduction of Wise-IoU further increases the performance of the model without affecting the complexity.

Finally, in terms of outcomes on the coco128 dataset, the ICDW-YOLO model emerges as the most exceptional overall, particularly with the precision and comprehensive performance of the model with complexity and accuracy ranking highest across all models. In comparison to the original YOLOv8 model, its precision, recall, mAP50, and mAP50–95 increased by 17.527%, 1.725%, 8.97%, and 4.583%. Additionally, on the Crack dataset, ICDW-YOLO ranked first in all metrics except recall, where it was only marginally behind model (e), which had a FLOP value nearly twice that of ICDW-YOLO. In comparison to the original YOLOv8 model, its precision, recall, mAP50, and mAP50–95 increased by 2.049%, 1.405%, 0.264%, and 4.392%, respectively.

#### 4.4.3. Quantitative Comparison and Evaluation

Additionally, after the quantitative analysis, this study delved into the detection performance of ICDW-YOLO through qualitative analysis. Initially, to evaluate the crack detection capabilities of ICDW-YOLO for wooden materials, as depicted in Figure 4d, sixteen images showcasing diverse crack patterns, textures, exposure levels, and blur degrees were extracted from the Crack dataset. ICDW-YOLO consistently yielded reliable outcomes for crack detection in wooden materials across varied and intricate conditions.

**Figure 5:**
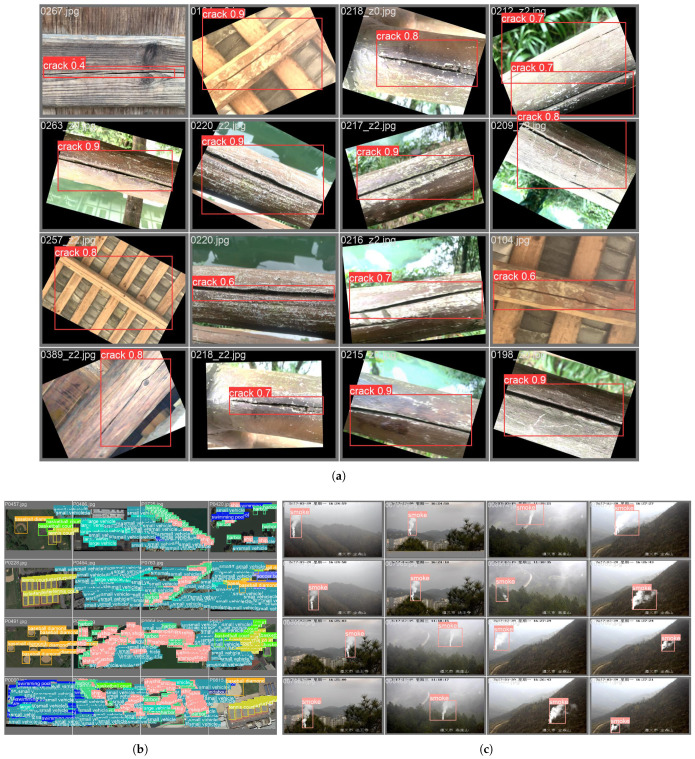
Target detection sample of ICDW-YOLO. (**a**) Examples of detection results on the Crack dataset. (**b**) Examples of detection results on the DOTAv2-tiny dataset. (**c**) Examples of detection results on the Fire and Smoke dataset.

Moreover, to assess the model’s ability to generalize across different scenarios, as illustrated in Figure 5a,b, sixteen aerial images featuring multiple targets and small objects from the DOTAv2-tiny dataset were selected. Additionally, sixteen images depicting more minor, abrupt, and dynamically evolving smoke plumes induced by wildfires were chosen from the Fire and Smoke dataset. It was noted that ICDW-YOLO not only delivered dependable results for detecting multiple targets and small objects but also exhibited commendable performance in discerning dynamically changing small objects.

Overall, the dataset proposed in this study constitutes a high-quality resource for crack detection in wooden materials. The ICDW-YOLO model proposed herein demonstrates robustness in detecting objects of varying sizes and compositions, encompassing single and multiple objects, as well as those exhibiting dynamic changes. In the domain of crack detection in wooden materials, ICDW-YOLO adeptly identifies and detects cracks, thereby offering valuable support in the surveillance and preservation of ancient structures.

## 5. Conclusions

This research presents a refined crack detection algorithm tailored for wooden materials, leveraging enhancements made to YOLOv8. In contrast to YOLOv8n, the algorithmic framework proposed herein initially restructures the neck network by adopting the GD mechanism and replacing the C2f module with the ICDW layer. Subsequently, GSConv is introduced to supplant the convolutional process in the neck network. Wise-IoU is further integrated, which features a dual-layer attention mechanism and dynamic gradient gain attributes to better align with the intricacies of crack detection in wooden materials. Empirical findings demonstrate that the algorithm proposed in this study surpasses existing state-of-the-art algorithms in terms of detection accuracy and exhibits promising experimental outcomes on internally developed and publicly available datasets. Furthermore, it fortifies the equilibrium between algorithmic efficacy and intricacy while enhancing overall performance.

Despite achieving commendable detection accuracy, the proposed algorithm still encounters instances of missed detections and false alarms. The network’s resilience may diminish in response to alterations in lighting conditions. Our future research will prioritize the comprehensive collection of crack images in wooden materials and will further investigate the relationships among factors such as types of wooden materials, quality of data annotation, types of cracks, crack orientations, and their future development trends. Additionally, in-depth studies will be conducted on the impact of various types of cracks in wooden materials on the structural health of buildings. Additionally, the main objective of model research is to accelerate detection speed and robustness while keeping model complexity from significantly increasing, improving real-time detection performance, and integrating multiple data sources to enhance detection accuracy and reliability.

## Figures and Tables

**Table 1:** Description of the Crack dataset.

Items	Sample Count
Training set	1131
Validation set	324
Test set	162
Total	1617

**Table 2:** Descriptions of other datasets.

Dataset	Source	Training Set	Validation Set	Test Set
Fire and Smoke	Self-collection USTC [62]	720 6310	360 3155	360 3155
DOTAv2-tiny	DOTAv2 [63]	1521	1170	1334
coco128	coco128	128	128	128

**Table 3:** Descriptions of hardware and software.

Items	Descriptions
Storage	SSD: 2 TB (SAMSUNG, Seongnam si, Gyeonggi-do, Korea)
RAM	DDR5 32 GB (Kingston, Fountain Valley, CA, USA)
Motherboard	MPG Z790 EDGE TI MAX WIFI (MSI, New Taipei City, Taiwan, China)
GPU	GeForce 4060 Ti 16 GB (Nvidia, Santa Clara, CA, USA)
CPU	Intel core i7 processor 14,700 K (Intel, Santa Clara, CA, USA)
OS	Windows 11 Pro:22H2

**Table 4:** Hyperparameters for training.

Dataset	Train Hyperparameters	Details
Crack	Learning rate	0.001
	Batch size	16
	Epoch	200
	Image size	640
DATAv2-tiny	Learning rate	0.01
	Batch size	16
	Epoch	235
	Image size	640
Fire and Smoke	Learning rate	0.01
	Batch size	16
	Epoch	200
	Image size	640
coco128	Learning rate	0.001
	Batch size	32
	Epoch	980
	Image size	640

**Table 5:** Quantitative analysis index table for the model.

Dataset	Methods	Precision (%)	Recall (%)	mAP50 (%)	mAP50–95 (%)	F1 Score	FLOPs (G)	FPS
Crack	YOLOv5	96.331	96.608	98.937	74.626	0.965	7.1	526.32
	YOLOv5p6	96.291	97.211	98.785	74.831	0.968	7.2	526.32
	YOLOv6	96.839	96.605	99.018	76.708	0.967	11.8	555.56
	YOLOv8	97.133	97.413	98.925	77.149	0.973	8.1	238.10
	YOLOv8p6	97.260	97.222	98.897	77.014	0.972	8.1	250.00
	EfficientNetv2	95.328	97.222	98.250	73.688	0.963	2.6	666.67
	ShuffleNetV2	96.225	98.108	99.220	76.135	0.972	16.4	555.56
	RTDETR	7.09	45.072	1.298	0.282	0.123	103.4	105.26
	ICDW-YOLO	98.756	98.013	99.201	79.018	0.984	11.3	476.19
DATAv2-tiny	YOLOv5	72.929	42.129	46.798	25.701	0.534	7.1	294.12
	YOLOv5p6	73.501	39.293	44.575	24.788	0.512	7.2	263.15
	YOLOv6	58.660	39.996	42.639	25.221	0.476	11.8	232.56
	YOLOv8	80.667	41.841	46.749	28.457	0.551	8.1	312.50
	YOLOv8p6	71.745	44.795	46.658	26.697	0.552	8.1	322.58
	EfficientNetv2	42.969	20.368	22.601	11.539	0.276	2.6	400.00
	ShuffleNetV2	61.457	38.170	42.081	25.022	0.471	16.5	270.27
	RTDETR	48.637	28.232	29.628	16.222	0.357	103.8	78.74
	ICDW-YOLO	83.155	47.131	48.039	28.071	0.602	11.3	312.50
Fire and Smoke	YOLOv5	99.660	99.230	99.493	96.829	0.994	7.1	833.33
	YOLOv5p6	99.035	99.069	99.482	97.216	0.991	7.2	769.23
	YOLOv6	99.240	99.560	99.491	98.115	0.994	11.8	1000.00
	YOLOv8	99.297	99.492	99.496	97.638	0.994	8.1	909.09
	YOLOv8p6	99.538	99.153	99.493	97.572	0.993	8.1	769.23
	EfficientNetv2	99.001	99.069	99.476	94.372	0.990	2.6	434.78
	ShuffleNetV2	99.230	99.543	99.498	97.003	0.994	16.5	370.37
	RTDETR	99.890	100.00	99.500	92.092	0.999	103.4	114.94
	ICDW-YOLO	99.628	99.661	99.498	98.336	0.996	11.3	625.00
coco128	YOLOv5	72.939	44.856	53.621	33.163	0.555	7.7	208.33
	YOLOv5p6	59.307	48.741	52.280	31.472	0.535	7.8	172.41
	YOLOv6	75.355	52.659	61.281	45.616	0.620	13.0	217.39
	YOLOv8	64.601	53.456	60.256	39.627	0.585	8.7	277.78
	YOLOv8p6	76.884	49.272	58.160	41.239	0.601	8.7	196.08
	EfficientNetv2	60.075	14.884	19.492	11.973	0.239	2.7	357.14
	ShuffleNetV2	69.562	52.708	57.973	39.605	0.600	16.6	270.27
	RTDETR	65.520	66.249	75.097	62.543	0.659	103.8	104.17
	ICDW-YOLO	82.128	55.181	69.226	44.210	0.660	11.8	196.08

**Table 6:** Results of ablation experiments.

Dataset	Model	GD Mechanism	ICDW layer and GSConv	Wise-IoU	Precision (%)	Recall (%)	mAP50 (%)	mAP50–95 (%)	FLOPs (G)
coco128	YOLOv8				64.601	53.456	60.256	39.627	8.7
	(a)	✓			74.535	49.239	55.418	37.862	12.5
	(b)		✓		76.661	58.848	66.131	50.077	20.0
	(c)			✓	70.032	59.802	66.174	39.992	8.7
	(d)	✓	✓		74.361	51.338	57.961	39.670	11.8
	(e)		✓	✓	79.921	60.336	75.146	49.444	20.0
	Proposed framework	✓	✓	✓	82.128	55.181	69.226	44.210	11.8
Crack	YOLOv8				96.331	96.608	98.937	74.626	8.1
	(a)	✓			97.902	97.222	99.046	77.274	11.9
	(b)		✓		97.985	97.840	99.033	78.065	19.8
	(c)			✓	97.448	97.457	99.041	78.278	8.1
	(d)	✓	✓		97.266	97.296	98.596	78.757	10.0
	(e)		✓	✓	98.380	98.879	99.191	78.780	19.8
	Proposed framework	✓	✓	✓	98.756	98.013	99.201	79.018	11.3

## Data Availability

The data presented in this study are available upon request from the corresponding author.

## References

[1] Dai J., Chang L., Qian W., Li X. (2016). Damage characteristics of ancient architecture wood members and stress wave nondestructive testing of internal void. J. Beijing Univ. Technol..

[2] Cabaleiro M., Lindenbergh R., Gard W., Arias P., Van de Kuilen J. (2017). Algorithm for automatic detection and analysis of cracks in timber beams from LiDAR data. Constr. Build. Mater..

[3] Yang H., Yu L. (2017). Feature extraction of wood-hole defects using wavelet-based ultrasonic testing. J. For. Res..

[4] Kato S., Wada N., Shiogai K., Tamaki T., Kagawa T., Toyosaki R., Nobuhara H. (2023). Crack Severity Classification from Timber Cross-Sectional Images Using Convolutional Neural Network. Appl. Sci..

[5] Hadiwidjaja M.L., Gunawan P.H., Prakasa E., Rianto Y., Sugiarto B., Wardoyo R., Damayanti R., Sugiyanto K., Dewi L.M., Astutiputri V.F. (2019). Developing Wood Identification System by Local Binary Pattern and Hough Transform Method. J. Phys. Conf. Ser..

[6] Chang L.H., Chang X.H., Chang H., Qian W., Cheng L.T., Han X.L. (2019). Nondestructive testing on ancient wooden components based on Shapley value. Advances in Materials Science and Engineering.

[7] Hacıefendioğlu K., Ayas S., Başağa H.B., Toğan V., Mostofi F., Can A. (2022). Wood construction damage detection and localization using deep convolutional neural network with transfer learning. Eur. J. Wood Wood Prod..

[8] Ehtisham R., Qayyum W., Camp C.V., Plevris V., Mir J., Khan Q.U.Z., Ahmad A. (2024). Computing the characteristics of defects in wooden structures using image processing and CNN. Autom. Constr..

[9] Liu Y., Hou M., Li A., Dong Y., Xie L., Ji Y. (2020). Automatic Detection of Timber-Cracks in Wooden Architectural Heritage Using YOLOv3 Algorithm. Int. Arch. Photogramm. Remote Sens. Spat. Inf. Sci..

[10] Ma J., Yan W., Liu G., Xing S., Niu S., Wei T. (2022). Complex texture contour feature extraction of cracks in timber structures of ancient architecture based on YOLO algorithm. Adv. Civ. Eng..

[11] Li L., Li Z., Han H., Yang L., Feng X., Roli F., Xia Z. (2023). Wooden spoon crack detection by prior knowledge-enriched deep convolutional network. Eng. Appl. Artif. Intell..

[12] Qiu Y., Ai Z., Lin Y., Xu Z., Liu X. (2022). Detecting Defects of Wooden Boards by Improved YOLOv4-Tiny Algorithm. Proceedings of the 2021 Chinese Intelligent Systems Conference: Volume III.

[13] Wang B., Yang C., Ding Y., Qin G. (2021). Detection of wood surface defects based on improved YOLOv3 algorithm. BioResources.

[14] Lin Y., Xu Z., Chen D., Ai Z., Qiu Y., Yuan Y. (2023). Wood Crack Detection Based on Data-Driven Semantic Segmentation Network. IEEE/CAA J. Autom. Sin..

[15] Cao X., Li G. An effective method of wood crack trace and quantity detection based on digital image processing technology. Proceedings of the 2021 13th International Conference on Machine Learning and Computing.

[16] Redmon J., Divvala S., Girshick R., Farhadi A. You only look once: Unified, real-time object detection. Proceedings of the IEEE Conference on Computer Vision and Pattern Recognition.

[17] Jocher G. (2023). YOLOv8. Ultralytics: Github. https://github.com/ultralytics/ultralytics.

[18] Li H., Li J., Wei H., Liu Z., Zhan Z., Ren Q. (2024). Slim-neck by GSConv: A lightweight-design for real-time detector architectures. J. Real Time Image Process..

[19] Wang C., He W., Nie Y., Guo J., Liu C., Wang Y., Han K. (2024). Gold-YOLO: Efficient object detector via gather-and-distribute mechanism. Advances in Neural Information Processing Systems.

[20] Tong Z., Chen Y., Xu Z., Yu R. (2023). Wise-IoU: Bounding box regression loss with dynamic focusing mechanism. arXiv.

[21] Viola P., Jones M. (2001). Rapid object detection using a boosted cascade of simple features. Proceedings of the 2001 IEEE Computer Society Conference on Computer Vision and Pattern Recognition, CVPR 2001.

[22] Dalal N., Triggs B. (2005). Histograms of oriented gradients for human detection. Proceedings of the 2005 IEEE Computer Society Conference on Computer Vision and Pattern Recognition (CVPR’05).

[23] Wang X., Han T.X., Yan S. (2009). An HOG-LBP human detector with partial occlusion handling. Proceedings of the 2009 IEEE 12th International Conference on Computer Vision.

[24] Felzenszwalb P., McAllester D., Ramanan D. (2008). A discriminatively trained, multiscale, deformable part model. Proceedings of the 2008 IEEE Conference on Computer Vision and Pattern Recognition.

[25] Li J., Wong H.C., Lo S.L., Xin Y. (2018). Multiple object detection by a deformable part-based model and an R-CNN. IEEE Signal Process. Lett..

[26] Ye S., Zhou K., Zain A.M., Wang F., Yusoff Y. (2023). A modified harmony search algorithm and its applications in weighted fuzzy production rule extraction. Front. Inf. Technol. Electron. Eng..

[27] Qin F., Zain A.M., Zhou K.Q. (2022). Harmony search algorithm and related variants: A systematic review. Swarm Evol. Comput..

[28] Ye S.Q., Zhou K.Q., Zhang C.X., Mohd Zain A., Ou Y. (2022). An improved multi-objective cuckoo search approach by exploring the balance between development and exploration. Electronics.

[29] Zhang C.X., Zhou K.Q., Ye S.Q., Zain A.M. (2021). An Improved Cuckoo Search Algorithm Utilizing Nonlinear Inertia Weight and Differential Evolution for Function Optimization Problem. IEEE Access.

[30] Zhang X.Y., Zhou K.Q., Li P.C., Xiang Y.H., Zain A.M., Sarkheyli-Hägele A. (2022). An improved chaos sparrow search optimization algorithm using adaptive weight modification and hybrid strategies. IEEE Access.

[31] Lowe D.G. (1999). Object recognition from local scale-invariant features. Proceedings of the Seventh IEEE International Conference on Computer Vision.

[32] Rublee E., Rabaud V., Konolige K., Bradski G. (2011). ORB: An efficient alternative to SIFT or SURF. Proceedings of the 2011 International Conference on Computer Vision.

[33] Felzenszwalb P.F., Girshick R.B., McAllester D., Ramanan D. (2009). Object detection with discriminatively trained part-based models. IEEE Trans. Pattern Anal. Mach. Intell..

[34] Xiao L., Liao B., Li S., Chen K. (2018). Nonlinear recurrent neural networks for finite-time solution of general time-varying linear matrix equations. Neural Netw..

[35] Zhang Z., Zheng L., Weng J., Mao Y., Lu W., Xiao L. (2018). A new varying-parameter recurrent neural-network for online solution of time-varying Sylvester equation. IEEE Trans. Cybern..

[36] Xiao L., Liao B. (2016). A convergence-accelerated Zhang neural network and its solution application to Lyapunov equation. Neurocomputing.

[37] Liao B., Zhang Y. (2013). Different complex ZFs leading to different complex ZNN models for time-varying complex generalized inverse matrices. IEEE Trans. Neural Netw. Learn. Syst..

[38] Liao B., Zhang Y. (2014). From different ZFs to different ZNN models accelerated via Li activation functions to finite-time convergence for time-varying matrix pseudoinversion. Neurocomputing.

[39] Jin L., Zhang Y., Li S., Zhang Y. (2016). Modified ZNN for Time-Varying Quadratic Programming With Inherent Tolerance to Noises and Its Application to Kinematic Redundancy Resolution of Robot Manipulators. IEEE Trans. Ind. Electron..

[40] Zhang Y., Li S., Kadry S., Liao B. (2018). Recurrent neural network for kinematic control of redundant manipulators with periodic input disturbance and physical constraints. IEEE Trans. Cybern..

[41] Liu W., Anguelov D., Erhan D., Szegedy C., Reed S., Fu C.Y., Berg A.C. (2016). SSD: Single shot multibox detector. Proceedings of the Computer Vision—ECCV 2016: 14th European Conference.

[42] Krizhevsky A., Sutskever I., Hinton G.E. (2017). ImageNet classification with deep convolutional neural networks. Commun. ACM.

[43] He K., Zhang X., Ren S., Sun J. Deep residual learning for image recognition. Proceedings of the IEEE Conference on Computer Vision and Pattern Recognition.

[44] Huang G., Liu Z., Van Der Maaten L., Weinberger K.Q. Densely connected convolutional networks. Proceedings of the IEEE Conference on Computer Vision and Pattern Recognition.

[45] Howard A.G., Zhu M., Chen B., Kalenichenko D., Wang W., Weyand T., Andreetto M., Adam H. (2017). Mobilenets: Efficient convolutional neural networks for mobile vision applications. arXiv.

[46] Zhang X., Zhou X., Lin M., Sun J. Shufflenet: An extremely efficient convolutional neural network for mobile devices. Proceedings of the IEEE Conference on Computer Vision and Pattern Recognition, Salt Lake City.

[47] Ma N., Zhang X., Zheng H.T., Sun J. Shufflenet v2: Practical guidelines for efficient cnn architecture design. Proceedings of the European Conference on Computer Vision (ECCV).

[48] Chen J., Wang W., Zhang D., Zeb A., Nanehkaran Y.A. (2021). Attention embedded lightweight network for maize disease recognition. Plant Pathol..

[49] Jocher G. (2020). YOLOv5. Ultralytics: Github. https://github.com/ultralytics/yolov5.

[50] Wang C.Y., Bochkovskiy A., Liao H.Y.M. YOLOv7: Trainable Bag-of-Freebies Sets New State-of-the-Art for Real-Time Object Detectors. Proceedings of the 2023 IEEE/CVF Conference on Computer Vision and Pattern Recognition (CVPR).

[51] Redmon J., Farhadi A. (2018). YOLOv3: An Incremental Improvement. arXiv.

[52] He K., Zhang X., Ren S., Sun J. (2015). Spatial pyramid pooling in deep convolutional networks for visual recognition. IEEE Trans. Pattern Anal. Mach. Intell..

[53] Liu S., Qi L., Qin H., Shi J., Jia J. Path aggregation network for instance segmentation. Proceedings of the IEEE Conference on Computer Vision and Pattern Recognition.

[54] Li X., Wang W., Wu L., Chen S., Hu X., Li J., Tang J., Yang J. (2020). Generalized focal loss: Learning qualified and distributed bounding boxes for dense object detection. Adv. Neural Inf. Process. Syst..

[55] Wei K., Li J., Ma C., Ding M., Wei S., Wu F., Chen G., Ranbaduge T. (2022). Vertical federated learning: Challenges, methodologies and experiments. arXiv.

[56] Zheng Z., Wang P., Liu W., Li J., Ye R., Ren D. Distance-IoU loss: Faster and better learning for bounding box regression. Proceedings of the AAAI Conference on Artificial Intelligence.

[57] Feng C., Zhong Y., Gao Y., Scott M.R., Huang W. (2021). Tood: Task-aligned one-stage object detection. Proceedings of the 2021 IEEE/CVF International Conference on Computer Vision (ICCV).

[58] Lin T.Y., Dollár P., Girshick R., He K., Hariharan B., Belongie S. Feature pyramid networks for object detection. Proceedings of the IEEE Conference on Computer Vision and Pattern Recognition.

[59] Ye M., Wang Z., Lan X., Yuen P.C. Visible thermal person re-identification via dual-constrained top-ranking. Proceedings of the IJCAI.

[60] Liu B., Wang M., Foroosh H., Tappen M., Pensky M. Sparse convolutional neural networks. Proceedings of the IEEE Conference on Computer Vision and Pattern Recognition.

[61] Chollet F. Xception: Deep learning with depthwise separable convolutions. Proceedings of the IEEE Conference on Computer Vision and Pattern Recognition.

[62] Zhang Q., Lin G., Zhang Y., Xu G., Wang J. (2018). Wildland Forest Fire Smoke Detection Based on Faster R-CNN using Synthetic Smoke Images. Procedia Eng..

[63] Xia G.S., Bai X., Ding J., Zhu Z., Belongie S., Luo J., Datcu M., Pelillo M., Zhang L. DOTA: A large-scale dataset for object detection in aerial images. Proceedings of the IEEE Conference on Computer Vision and Pattern Recognition.

[64] Li C., Li L., Jiang H., Weng K., Geng Y., Li L., Ke Z., Li Q., Cheng M., Nie W. (2022). YOLOv6: A single-stage object detection framework for industrial applications. arXiv.

[65] Tan M., Le Q. Efficientnet: Rethinking model scaling for convolutional neural networks. Proceedings of the International Conference on Machine Learning. PMLR.

[66] Zhao Y., Lv W., Xu S., Wei J., Wang G., Dang Q., Liu Y., Chen J. Detrs beat yolos on real-time object detection. Proceedings of the IEEE/CVF Conference on Computer Vision and Pattern Recognition.

